# Pyrethroids Toxicity to Male Reproductive System and Offspring as a Function of Oxidative Stress Induction: Rodent Studies

**DOI:** 10.3389/fendo.2021.656106

**Published:** 2021-05-27

**Authors:** Xu Zhang, Tongtong Zhang, Xiaohan Ren, Xinglin Chen, ShangQian Wang, Chao Qin

**Affiliations:** The State Key Lab of Reproductive, Department of Urology, The First Affiliated Hospital of Nanjing Medical University, Nanjing, China

**Keywords:** pyrethroids, meta-analysis, sperm performance, fertility, male reproduction

## Abstract

Pyrethroids may be related to male reproductive system damage. However, the results of many previous studies are contradictory and uncertain. Therefore, a systematic review and a meta-analysis were performed to assess the relationship between pyrethroid exposure and male reproductive system damage. A total of 72 articles were identified, among which 57 were selected for meta-analysis, and 15 were selected for qualitative analysis. Pyrethroid exposure affected sperm count (SMD= -2.0424; 95% CI, -2.4699 to -1.6149), sperm motility (SMD=-3.606; 95% CI, -4.5172 to -2.6948), sperm morphology (SMD=2.686; 95% CI, 1.9744 to 3.3976), testis weight (SMD=-1.1591; 95% CI, -1.6145 to -0.7038), epididymal weight (SMD=-1.1576; 95% CI, -1.7455 to -0.5697), and serum testosterone level (SMD=-1.9194; 95% CI, -2.4589 to -1.3798) in the studies of rats. We found that gestational and lactational exposure to pyrethroids can reduce sperm count (SMD=1.8469; 95% CI, -2.9010 to -0.7927), sperm motility (SMD=-2.7151; 95% CI, -3.9574 to -1.4728), testis weight (SMD=-1.4361; 95% CI, -1.8873 to -0.9848), and epididymal weight (SMD=-0.6639; 95% CI, -0.9544 to -0.3733) of F1 offspring. Exposure to pyrethroids can increase malondialdehyde (SMD=3.3451; 95% CI 1.9914 to 4.6988) oxide in testes and can reduce the activities of glutathione (SMD=-2.075; 95% CI -3.0651 to -1.0848), superoxide dismutase (SMD=-2.4856; 95% CI -3.9612 to -1.0100), and catalase (SMD=-2.7564; 95% CI -3.9788 to -1.5340). Pyrethroid exposure and oxidative stress could damage male sperm quality. Gestational and lactational pyrethroid exposure affects the reproductive system of F1 offspring.

## Introduction

Synthetic pyrethroids (SPs) are among the most extensively used pesticides worldwide. They are derived from pyrethrins, which are found in the flowers of *Chrysanthemum cinerariaefolium* (1). In 1949, allethrin was identified as the first pyrethroid pesticide (2). With the continuous progress of science and technology, the number of SPs that have been developed has increased, some of which include: cypermethrin, deltamethrin, fenvalerate, permethrin, pyrethrin, resmethrin, and sumithrin (3). Pyrethroids are endocrine-disrupting chemicals (EDCs) that are responsible for male reproductive impairment (4). According to their chemical structure, pyrethroids consist of two types. Type I pyrethroids do not have a cyano moiety at the α-position (e.g., permethrin and allethrin), whereas Type II pyrethroids, such as cypermethrin, have α-cyano moiety (5). With the phasing out of the varieties of organochlorine and organophosphorus pesticides, pyrethroids have been widely applied to agricultural production, household use, and public health places (6). Pyrethroids often show more advantages than traditional insecticides due to their increased environmental stability and toxicity toward insects (7).

In the past two decades, people have been increasingly exposed to SPs in the environment and homes and through their diet due to the extensive use of these SPs. Acute toxic doses of type I pyrethroids can cause hyperexcitation, ataxia, tremor, and paralysis, whereas type II pyrethroids can lead to hypersensitivity, salivation, and choreoathetosis (8). In recent years, especially since pyrethroids have been listed as direct or indirect endocrine disruptors, an increasing number of studies have focused on their reproductive and endocrine risks. A variety of pyrethroids and their metabolites may disrupt hormone receptors and further interfere with the endocrine reproductive system (9, 10). A recent study showed that workers exposed to pyrethroids had poor semen quality (11).

Many studies have been conducted on mammals. Exposure to cypermethrin was associated with decreased levels of endocrine hormones, such as testosterone, luteinizing hormone, and follicle-stimulating hormone, as well as the testis and epididymis weights of rats (12). E. Kilian et al. focused on the effect of deltamethrin and phytoestrogens on rat reproductive parameters, and the results suggested that they both influenced sperm count and testis mass; the authors hypothesized that deltamethrin may have estrogenic effects (13, 14).

We conducted a systematic review and aggregated the available published data on the effect of SPs on semen parameters using a meta-analysis. Our study aims to determine the influence of SPs on the reproductive parameters of humans and rats and potential toxic effects on offspring.

## Materials and Methods

### Search Strategy

On March 24, 2021, we conducted systematic searches in PubMed, EMBASE, and Web of Science to identify all relevant studies published from 1990 to March 2021 using the following search terms: ((((((((((((((((((((((((((pyrethrin) OR pyrethroid*) OR allethrin stereoisomers) OR bifenthrin) OR beta-cyfluthrin) OR cyfluthrin) OR cyphenothrin) OR deltamethrin) OR esfenvalerate) OR etofenprox) OR fenpropathrin) OR taufluvalinate) OR lambda cyhalothrin) OR gamma cyhalothrin) OR imiprothrin) OR 1RS cispermethrin) OR permethrin) OR prallethrin) OR resmethrin) OR sumithrin) OR d-phenothrin) OR tefluthrin) OR tralomethrin) OR zeta-cypermethrin) OR cypermethrin) OR tetramethrin) AND ((((((((((((((sperm count) OR (sperm concentration)) OR (sperm morphology)) OR (sperm motility)) OR (sperm energy metabolism)) OR (sperm viability)) OR (sperm fertilization capacity)) OR (sperm capacitation reaction)) OR (sperm acrosome reaction)) OR (follicle stimulating hormone)) OR (luteinizing hormone)) OR (testosterone, estrogen)) OR (testis size)) OR (testis weight)) OR (sexual drive). A total of 307, 146, and 337 results were respectively retrieved in PubMed, EMBASE, and Web of Science. After removing duplicates, we were left with 509 potentially relevant articles.

The retrieved titles and abstracts were initially screened. Full texts of selected abstracts matching the inclusion criteria were obtained.

### Study Selection and Eligibility Criteria

We included animal and human studies from which primary data were gathered. The titles and abstracts of retrieved articles were independently screened by at least three of the authors (XZ, TZ, and XR). Articles deemed potentially eligible by either reviewer were retrieved for full-text review. The following inclusion and exclusion criteria were used for the meta-analysis. (1) The article should have been published between January 1990 and June 2020. (2) Pyrethroid pesticide should be the only insecticide used in the experiment, which did not include other insecticides, such as dichlorodiphenyltrichloroethane (DDT) and parathion. (3) Animal studies that involved mice or rats were included. (4) Studies involving the combination of pyrethroid pesticide and other insecticide were also excluded. (5) The paper should report the reproductive parameters, such as sperm count, sperm motility, sperm morphology, or serum testosterone. (6) Appropriate result data must be included, and the mean values and standard deviation (Mean ± SD) were used to perform quantitative analysis (standard error is transformed into standard deviation). We identified 57 studies that met our inclusion criteria (15–71).

### Data Extraction and Quality Assessment

To minimize bias and improve reliability, three researchers (XZ, TZ, and XR) independently extracted the data and resolved disagreements by discussion. In addition to the data on the means and standard deviations of relative sperm and testis parameters with and without pyrethroid pesticide exposure, the following data were also extracted: first author, dates of publication, the country of publication, published year, strains of rats, exposure periods, and dose. We used Engauge Digitizer to extract information from figures if no other statistical estimates were available. After the extraction of data, they were checked by the authors for discrepancies to minimize the possibility of errors.

### Data Synthesis and Analysis

All statistical analyses were carried out in R version 3.6.3 (R Foundation for Statistical Computing, Vienna, Austria; http://www.R-project.org). Pooled standardized mean difference (SMD) between comparison groups were calculated to determine the effect size. Both fixed effects models and random effects models (REMs) were fitted to assess the model types that were most suited to the data. Heterogeneity was evaluated using the Q test and the I2 statistic. Statistical significance was set at a p value <0.05. Publication bias was assessed using funnel plots for direct comparisons with 10 or more studies (**Appendix B** in **Supplementary Material**). Sensitivity analysis was conducted to evaluate the influence of individual studies on the summary effect estimate.

## Results

### Study Base

A total of 57 studies were included in the meta-analysis, including 20 rat studies and 37 mice studies. The number of studies included in each meta-analysis varied according to the sperm parameters reported, as follows: 31 studies provided data on sperm count; 25 studies provided data on motility; 28 studies provided data on morphology; 21 provided data on testis weight; 22 provided data on epididymis weight; and 22 provided data on serum testosterone. In the following sections, we first present the results of the quantitative meta-analysis. Next, we review papers that did not report results.

### Global Assessment: Reproductive Toxicity of All Pyrethroid Insecticides in Rats and Mice

**Figure 1:**
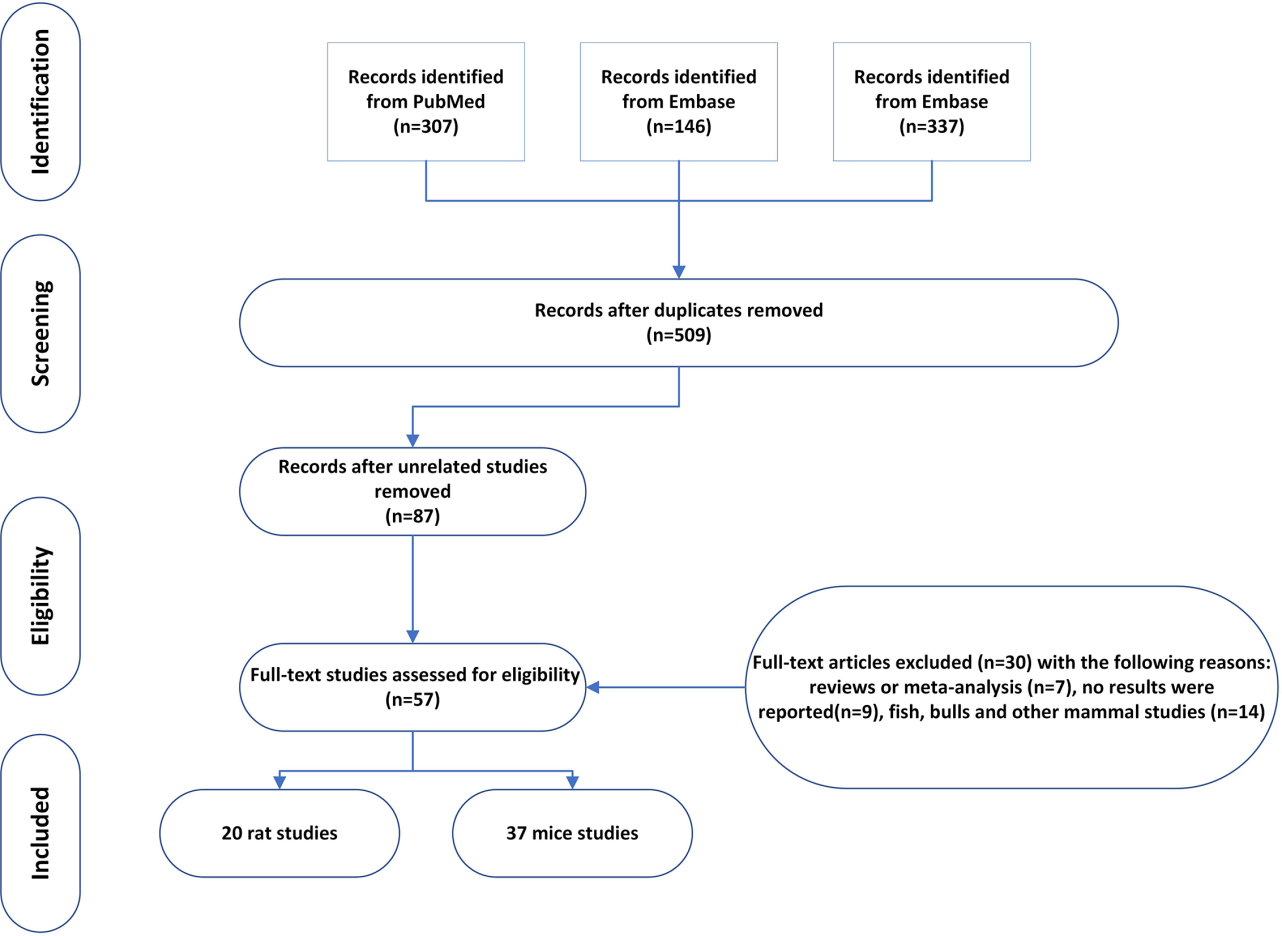
Study Selection Flow Diagram.

**Figure 2:**
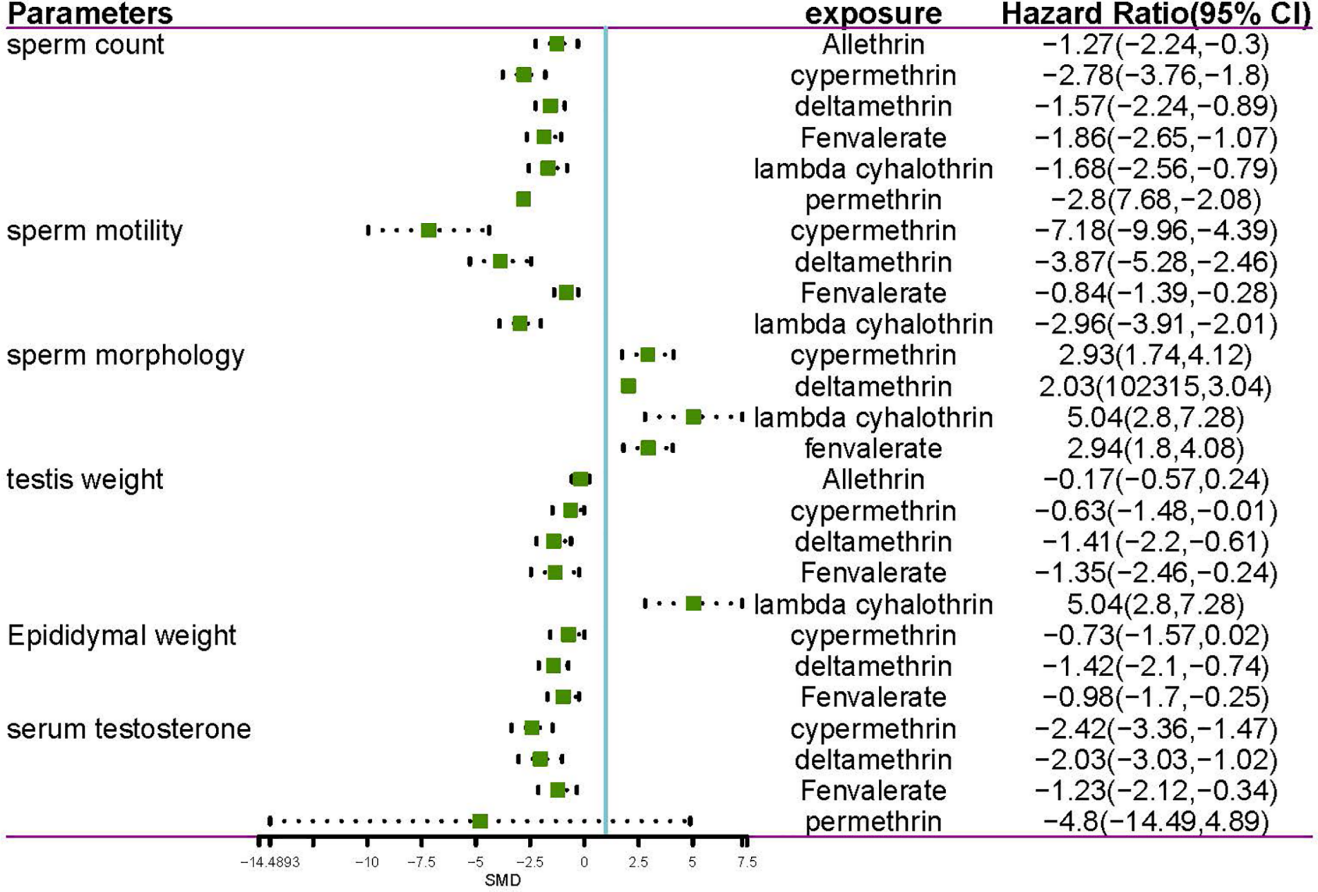
Forest plot showing the effect of pyrethroid exposure on rats.

**Figure 3:**
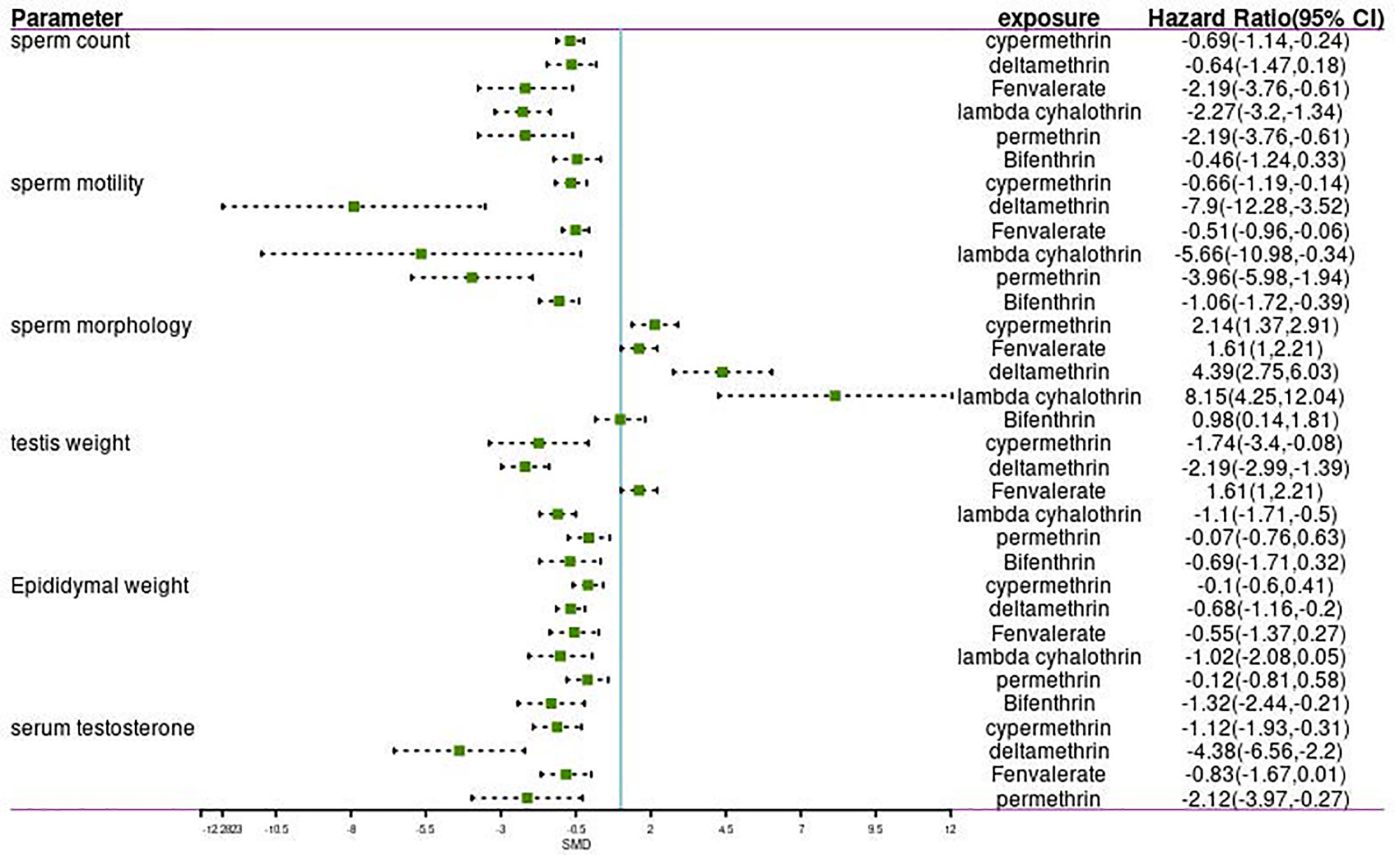
Forest plot showing the effect of pyrethroid exposure on mice.

The meta-analysis gives equal weight to each of the xenobiotic exposure indicators, including allethrin, bifenthrin, cypermethrin, deltamethrin, fenvalerate, lambda-cyhalothrin, and permethrin. Statistics were obtained, and analysis was performed on rats and mice, respectively (**Tables 1**, **2** and **Figures 1**–**3**). The funnel plots showed that the study had a risk of bias (**Appendix B** in the **Supplementary Material**).

**Table 1:** The effect of pyrethroid exposure on rats.

exposure	outcome	N studies	N estimates (min-max per study)	SMD	95% CI		I^2
all	sperm count	18	44	-2.0424	-2.4699	-1.6149	83.40%
	sperm motility	12	23	-3.606	-4.5172	-2.6948	87.70%
	sprem morphology	9	9	2.686	1.9744	3.3976	77.80%
	testis weight	13	29	-1.1591	-1.6145	-0.7038	81.20%
	epididymis weight	14	5	-1.1576	-1.7455	-0.5697	74.90%
	Serum testosterone	15	30	-1.9194	-2.4589	-1.3798	81.50%
Allethrin	sperm count	1	4	-1.2696	-2.2407	-0.2984	39.40%
	testis weight	1	4	-0.1667	-0.5693	0.236	0.00%
cypermethrin	sperm count	4	12	-2.4081	-3.2379	-1.5783	76.50%
	sperm motility	4	6	-5.9463	-8.4215	-3.4712	76.50%
	sprem morphology	3	3	2.9312	1.7422	4.1202	16.90%
	testis weight	5	9	-0.5881	-1.3006	0.1244	75.80%
	epididymis weight	4	7	-0.647	-1.4221	0.1282	74.50%
	Serum testosterone	7	12	-2.4153	-3.3608	-1.4697	80.70%
deltamethrin	sperm count	3	5	-1.3369	-1.9301	-0.7437	58.80%
	sperm motility	5	9	-4.1547	-5.7526	-2.5567	90.10%
	sprem morphology	3	5	1.9273	0.9292	2.9254	82.30%
	testis weight	4	8	-1.4087	-2.203	-0.6143	79.50%
	epididymis weight	2	5	-1.419	-2.0986	-0.7393	28.40%
	Serum testosterone	5	9	-2.0261	-3.0314	-1.0209	86.10%
Fenvalerate	sperm count	4	15	-1.859	-2.6511	-1.0668	89.80%
	sperm motility	1	4	-0.8359	-1.3942	-0.2775	29.30%
	testis weight	2	5	-1.3504	-2.4633	-0.2374	81.20%
	epididymis weight	1	2	-0.9764	-1.702	-0.2508	0.00%
	Serum testosterone	3	7	-1.2312	-2.1237	-0.3387	71.80%
lambda cyhalothrin	sperm count	1	2	-1.676	-2.5584	-0.7935	23.40%
	sperm motility	1	2	-2.9581	-3.911	-2.0052	0
	sprem morphology	1	1	5.0385	2.8016	7.2753	
permethrin	sperm count	2	2	-2.7997	7.6751	2.0756	89.90%
	testis weight	2	2	-7.7276	-10.5124	-4.9427	0
	Serum testosterone	2	2	-4.8016	-14.4893	4.8862	92.90%

**Table 2:** The effect of pyrethroid exposure on mice.

exposure	outcome	N studies	N estimates (min-max per study)	SMD	95% CI		I^2
all	sperm count	13	20	-1.836	-2.4656	-1.2064	82.00%
	sperm motility	13	26	-2.6366	-3.4075	-1.8658	86.60%
	sprem morphology	11		2.6804	2.0889	3.2719	81.30%
	testis weight	8	13	-1.0407	-1.6251	-0.4563	75.80%
	epididymis weight	8	5	-0.3717	-0.702	-0.0414	0.00%
	Serum testosterone	7	12	-1.5716	-2.2832	-0.8599	80.60%
cypermethrin	sperm count	3	5	-0.6905	-1.1433	-0.2377	20.10%
	sperm motility	1	3	-0.6646	-1.1916	-0.1376	0
	sprem morphology	1	6	2.1365	1.3652	2.9079	85.50%
	testis weight	2	4	-1.7379	-3.3954	-0.0805	89.20%
	epididymis weight	1	3	-0.0957	-0.6022	0.4108	0
	Serum testosterone	3	6	-1.1214	-1.9308	-0.3121	76.70%
deltamethrin	sperm count	1	1	-0.642	-1.4665	0.1824	
	sperm motility	5	5	-7.9005	-12.2823	-3.5186	93.90%
	sprem morphology	6	8	4.3899	2.7453	6.0344	82.40%
	testis weight	1	1	-2.1879	-2.9873	-1.3884	
	epididymis weight	3	5	-0.6766	-1.1582	-0.195	47.60%
	Serum testosterone	1	1	-4.3819	-6.5644	-2.1993	
Fenvalerate	sperm count	1	4	-2.1858	-3.7627	-0.6089	81.30%
	sperm motility	1	4	-0.509	-0.9572	-0.0609	0
	sprem morphology	1	4	1.6084	1.0044	2.2124	23.80%
	testis weight	1	1	0	-0.8002	0.8002	
	epididymis weight	1	1	-0.5517	-1.3699	0.2664	
	Serum testosterone	1	1	-0.8264	-1.6664	0.0136	
lambda cyhalothrin	sperm count	1	3	-2.2729	-3.2037	-1.3421	0
	sperm motility	1	3	-5.6613	-10.9837	-0.3389	88.10%
	sprem morphology	1	3	8.1474	4.2507	12.044	58.90%
	testis weight	2	4	-1.1035	-1.7082	-0.4988	0
	epididymis weight	1	1	-1.0152	-2.0756	0.0452	
permethrin	sperm count	1	4	-2.1858	-3.7627	-0.6089	81.30%
	sperm motility	3	5	-3.9623	-5.9841	-1.9406	85.30%
	testis weight	1	2	-0.0676	-0.7617	0.6264	0
	epididymis weight	1	2	-0.1152	-0.809	0.5786	0
	Serum testosterone	2	4	-2.122	-3.9711	-0.2729	87.20%
Bifenthrin	sperm count	1	1	-0.6748	-1.6911	0.3415	
	sperm motility	1	1	-0.8782	-1.9189	0.1626	
	sprem morphology	1	1	0.9363	-0.1125	1.9851	
	testis weight	1	1	-0.6947	-1.7132	0.3237	
	epididymis weight	1	1	-1.3229	-2.436	-0.2097	

#### Sperm Count

Among the works that involved rats, 18 studies and 44 estimates evaluated the effects of pyrethroids on sperm count (**Tables 1** and **2**). Pooled results indicated that sperm count was lower in pyrethroid exposure groups (REM SMD = -2.0424; 95% CI, -2.4699 to -1.6149; p<0.0001). In the works involving mice, 13 studies demonstrated the effects of pyrethroids on sperm count. Sperm count was significantly affected by pyrethroids (REM SMD = -1.836; 95% CI, -2.4656 to -1.2064; p<0.0001). Both sensitivity analyses demonstrated that the observed pooled effect size was not affected by the removal of any of the studies.

#### Sperm Motility

Twelve and thirteen studies were conducted on the toxic effects of pyrethroids on rats and mice, respectively (**Tables 1** and **2**). Sperm motility was lower in the pyrethroid exposure groups than in the control groups, and the pooled SMDs by REM were -3.606 (95% CI, -4.5172 to -2.6948; p < 0.0001) and -2.6366 (95% CI, -3.4075 to -1.8658 p < 0.0001), respectively. When any of the other studies were removed, the observed pooled effect size was not affected.

#### Sperm Morphology

Nine rat studies and eleven mice studies observed an abnormal rate in sperm morphology (**Tables 1** and **2**). The pooled results indicated that pyrethroid exposure was a risk factor of impaired morphology, and the SMD by REM was 2.686 (95% CI, 1.9744 to 3.3976; p < 0.0001) and 2.6804 (95% CI, 2.0889 to 3.2719; p < 0.0001) in rat and mice studies, respectively. Each sensitivity analysis was not affected by the removal of any of the studies.

#### Testis Weight and Epididymal Weight

In the studies of rats, the pooled SMD by REM of testis weight was -1.1591 (95% CI, -1.6145 to -0.7038 p < 0.0001), and the epididymal weight was also lower in the pyrethroid exposure groups than in the control groups. The pooled SMD by REM was -1.1576 (95% CI, -1.7455 to -0.5697; p = 0.0001). Similarly, in studies on mice, the pooled SMDs by REM of testis and epididymal weight were -1.0407 (95% CI, -1.6251 to -0.4563; p = 0.0005) and -0.3717 (95% CI, -0.7020 to -0.0414; p = 0.0274), respectively. Overall, pyrethroids were a risk factor for reduced testis weight and epididymal weight. In the studies on mice epididymal weight, sensitivity analyses indicated that the paper of Jing-Yi Hu et al. showed a slight increase in the SMD to -1.07 (**Tables 1** and **2**).

#### Serum Testosterone

Fifteen and seven studies detected the content of serum testosterone in pyrethroid exposure and control groups of rats and mice, respectively (**Tables 1** and **2**). Overall, the serum testosterone level was significantly affected by pyrethroids, and the pooled SMDs by REM were -1.9194 (95% CI, -2.4589 to -1.3798; p < 0.0001) and -1.5716 (95% CI, -2.2832 to -0.8599; p < 0.0001).

### Subgroup Analysis for the Effect of Different Pyrethroids on Reproductive Parameters

Data were available for substance-specific analyses of any of the reproductive parameters for the most frequently examined pyrethroids (allethrin, bifenthrin, cypermethrin, deltamethrin, fenvalerate, lambda-cyhalothrin, and permethrin). The results are summarized in **Tables 1** and **2**. **Figures 2** and **3** show the forest plot.

### Effects of Pyrethroid Exposure on Gestational and Lactational Aspects of Reproductive System

**Figure 4:**
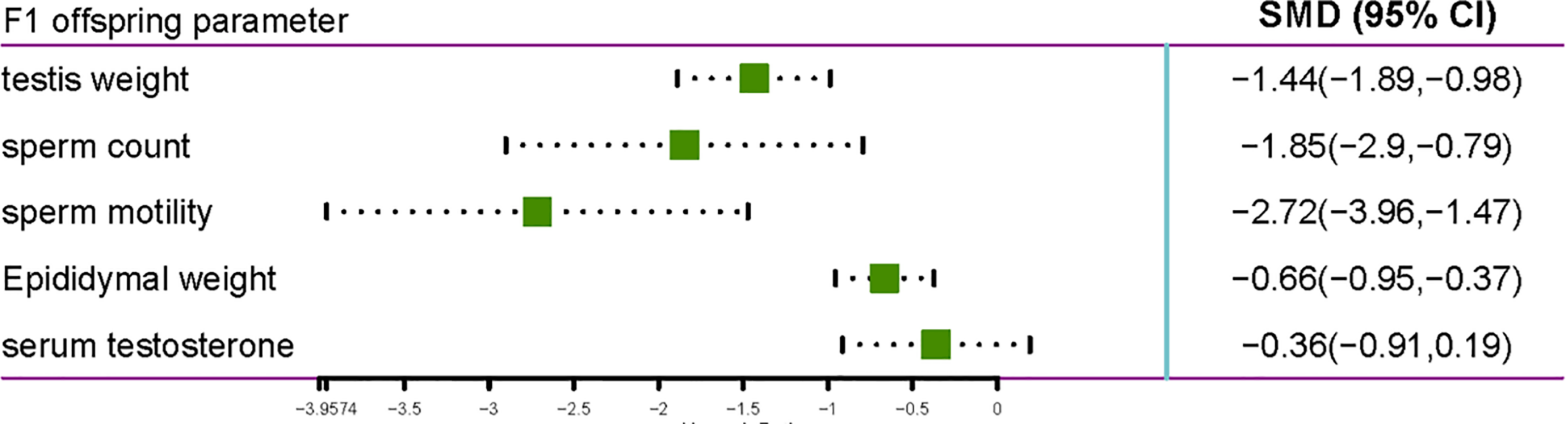
Forest plot showing the effect of pyrethroid exposure on the F1 offspring of rats and mice.

Eight studies that included 410 rats were included in this analysis (**Figure 4**). The studies reported various parameters, including sperm count, sperm motility, serum testosterone, testis weight, and epididymal weight, which were affected by gestational and lactational exposure to pyrethroids. In general, gestational and lactational pyrethroid exposure and F1 reproductive system were related, and the meta-analysis showed that the sperm count of F1 rats or mice decreased in the exposed groups (the pooled SMDs by REM was -1.8469; 95% CI, -2.9010 to -0.7927; p = 0.0006). Two studies examined the F1 sperm motility after gestational and lactational exposure to pyrethroids, and the SMD by REM was -2.7151 (95% CI, -3.9574 to -1.4728; p<0.0001). In addition, eight studies reported the decrease in testis weight, and the SMD by REM was -1.4361 (95% CI, -1.8873 to -0.9848; p<0.0001). Moreover, the epididymal weight showed a decreasing trend (REM SMD: -0.6639; 95% CI, -0.9544 to -0.3733; p<0.0001). However, the changes of serum testosterone were not significant (REM SMD: -0.3608; 95% CI, -0.9135 to 0.1919; p=0.2007).

### Pyrethroids Oxidative Stress Induction on Rats and Mice

**Figure 5:**
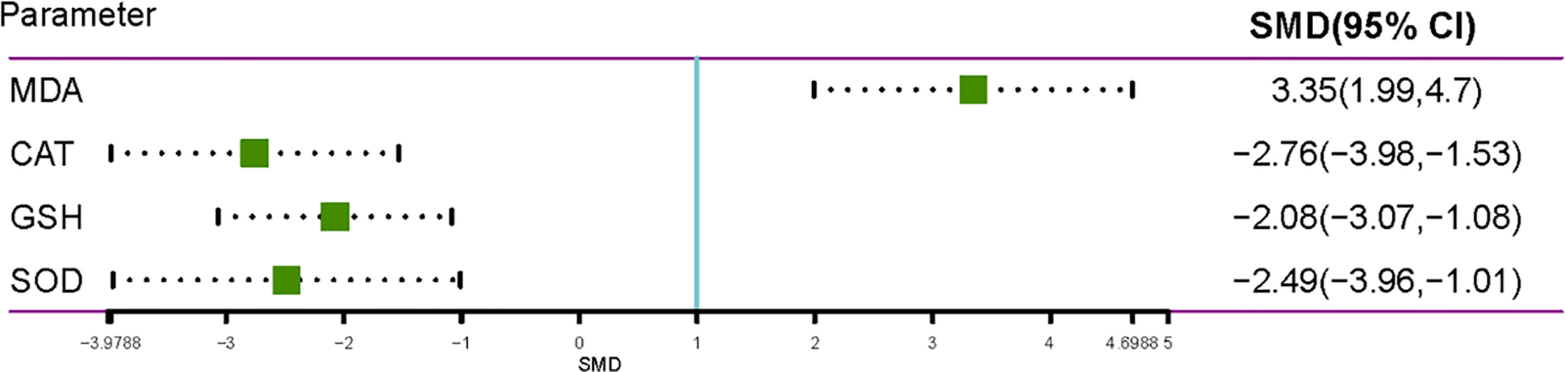
Forest plot showing the effect of pyrethroid oxidative stress induction on rats and mice.

Nine studies reported the presence of changes in the level of MDA, which is a product of lipid peroxidation, and testicular enzymatic activities (glutathione, GSH; superoxide dismutase, SOD; and catalase, CAT) of both the control and treated animals (**Figure 5**). Pyrethroids exposure significantly decreased the SOD, CAT, and GSH activities in the testis of treated animals compared with those of control animals, and the SMDs by REM were -2.4856 (95% CI -3.9612 to -1.0100), -2.7564 (95% CI -3.9788 to -1.5340), and -2.075 (95% CI -3.0651 to -1.0848), respectively. The MDA level increased compared with control animals, and the SMD by REM was 3.3451 (95% CI 1.9914 to 4.6988) (**Figure 5**).

### Human Research on the Effect of SPs About the Male Reproductive System

A total of 17 human studies were included in the analysis (**Table 3**), among which 15 studies evaluated the effect of pyrethroids on sperm quality. The details of these 17 studies are summarized in **Table 3**. Some urinary pyrethroid metabolites are 3-phenoxybenzoic acid (3-PBA) and cis- and trans-3-(2,2-dichlorovinyl)-2,2-dimethylcyclopropane carboxylic acid (CDCCA and TDCCA). 3-PBA is common in several pyrethroids, including cyhalothrin, cypermethrin, deltamethrin, fenvalerate, and permethrin, whereas CDCCA and TDCCA are metabolites of cis-permethrin and trans-permethrin, respectively.

**Table 3:** Characteristics of included human studies reporting an association between pyrethroid exposure and reproductive parameters.

Author	Year	Country	Mean age	Population	Exposure	Outcome	Conclusion
John D. Meeker	2008	America	35.7	207 Americans	Pyrethroids	Morphology concentration motility	Reduced semen quality and increased sperm DNA damage concerning urinary metabolites of pyrethroid insecticides
Michał Radwan	2014	Poland	32.2	334 men	Pyrethroids	Morphology concentration motility	Environmental pyrethroids exposure may affect semen quality and the level of reproductive hormones
Ramison Santos	2019	Brazil	45.6	75 men	Pyrethroids	FSH testosterone	Increase in male testosterone appeared to be the most significant effect of long-term pesticide exposure
Q Bian	2004	China	30.13 for exposure, 30.61 for control	21 exposure, 19 control	Fenvalerate	Concentration, motility, computer-assisted sperm analysis	Results indicated that occupational exposure to FE induced a significant increase in sperm DNA damage. FE exposure and percentage DNA in the tail, and positive sperm damage
J. Yoshinaga	2013	Japan	20.2	322 male university students in suburban Tokyo	Pyrethroids	FSH, testosterone, LH	There were no associations between urinary 3-PBA and serum hormone levels
A. Zalata	2013	Egypt		20 healthy normozoospermic men	Cypermethrin	Sperm velocity, sperm motility, sperm linear-velocity, sperm linearity-index, acrosin activity index, computer-assisted sperm analysis	Vitamins C and E are useful in improving the toxic effects of cypermethrin on spermatozoon
Yankai Xia	2004	China	26.92 for exposure, 28.62 for control	42 men	Fenvalerate	Semen volume, sperm concentration, sperm number per ejaculum, sperm motility, sperm abnormality, computer-assisted sperm analysis	Fenvalerate is one of the important genotoxic agents with potential genotoxicity to human sperm
Tan Lifeng	2006	China		32 male workers and 46 male administrators in the office	Fenvalerate	Sperm volume, sperm motility, sperm count, sperm concentration, sperm morphology, sperm movement ability	Fenvalerate hurt male workers’ semen quality in the study
Melissa J. Perry	2007	America		18 randomly selected urine samples	Pyrethroid	Sperm concentration	High prevalence of exposure to PYR pesticides and our preliminary analyses provided some suggestion that the higher exposure group had a lower sperm concentration
Yankai Xia	2008	China		376 men with nonobstructive infertility	Pyrethroid	Sperm volume, sperm concentration, sperm motility, sperm count	These observed associations between 3-PBA levels and some altered semen quality indicated the reproductive effects of pyrethroid exposure on adult men
John D	2009	America		161 men	Pyrethroid	FSH, LH, testosterone	Found evidence for increased gonadotropin levels, and decreased androgen and inhibin B levels
Guixiang Ji	2011	China	28.5	240 men	Pyrethroid	Seminal volume, sperm count, sperm concentration, sperm motility, sperm DNA fragmentation	Found evidence for both increased sperm DNA fragmentation and decreased sperm concentration, concerning the urinary concentration of 3-PBA among men from a clinical infertile population
Hiroki Toshima	2012	Japan	36.8	42 men	Pyrethroid	Sperm count, sperm concentration, sperm motility	This pilot study suggested the pyrethroid exposure level as a significant contributor to poorer semen quality
Joanna Jurewicz	2015	Poland	32.22	286 men	Pyrethroid	Sperm count, sperm concentration, sperm morphology	Results suggest that environmental pyrethroid exposure may affect sperm, DNA damage measures index indicated the reproductive effects of pyrethroid exposure on adult men
Michał Radwan	2015	Poland		195 men	Pyrethroid	Sperm volume, sperm concentration, sperm morphology	The results reported here found that pyrethroids may be a sperm aneugen
Joanna Jurewicz	2016	Poland	32.2	194 men	Pyrethroid	Sperm concentration, sperm motility, sperm morphology	Observed effects of a lower Y:X sperm chromosome ratio among men with concentrations of metabolites of synthetic pyrethroids in urine
Yi Hu	2020	China	31.16	346 men	Pyrethroid	Semen volume, sperm concentration, sperm count, sperm morphology, sperm motility	Environmental PYRs exposure might adversely affect semen quality in reproductive-age men

Several studies evaluated the relationship between the urinary pyrethroid metabolites and sperm quality. John D. Meeker et al. (72) studied the relationship between urinary pyrethroid metabolites and semen quality, sperm motion parameters, and sperm DNA damage in 207 men. The results showed the association between urinary metabolites of pyrethroid insecticides and the decrease in semen quality. In another article, Michał Radwan et al. (73) showed the significant associations between urinary pyrethroids metabolite levels and the decrease in sperm concentration, the level of testosterone, and the semen parameters, as determined by computer-aided analysis. In contrast, J. Yoshinaga (74) conducted an experiment involving 322 male university students and showed that no associations existed between urinary 3-PBA and serum hormone levels. A study of the relationship between the urinary metabolite of pyrethroid insecticides and semen quality conducted on 376 healthy participants focused on sperm quality (volume, motility, number of spermatozoa, and concentration), and results showed the association between urinary metabolite of pyrethroid insecticides and sperm quality (75). Furthermore, a high level of the urinary metabolite of pyrethroid was related to the increase in gonadotropin levels and decrease in androgen and inhibin B levels (76). A recent cross-sectional study of 346 men conducted by Yi Hu et al. (77) showed that the urinary metabolite of pyrethroids was negatively associated with sperm morphology, sperm count, and semen quality.

Only one study focused on pyrethroid exposure and the levels of reproductive hormones. Ramison Santos (78) assessed the association of short-term and long-term exposure to pesticides with circulating levels of reproductive hormones in an agricultural population in the South of Brazil. They found that recent use of lambda-cyhalothrin was associated with increased male luteinizing hormone (LH) levels.

An *in vitro* study (79) assessed the potential effect of cypermethrin on human spermatozoa and the possible ameliorative effects of vitamins C and E. Semen samples of 20 healthy normozoospermic men were used, and the results indicated that *in vitro* cypermethrin can alter sperm function and induce DNA damage in spermatozoa, which improved after the administration of vitamins C and E and was maximal when both vitamins were used together.

Several studies have reported the relationship between pyrethroids exposure and genetic or chromosome damage. A study focused on sperm DNA integrity; 240 healthy participants were recruited. Results showed a significant positive correlation between urinary 3-PBA level and sperm DNA fragmentation (80). A study by Yankai Xia et al. (81) investigated the possible association between fenvalerate and spermatozoa DNA damage. Nineteen fenvalerate-exposed workers and 23 non-exposed workers were included. Sperm DNA strands were found to have breaks in fenvalerate-exposed workers using the Comet and TUNEL assays. Another study aimed to assess the relationship between pyrethroid exposure and sperm DNA damage (82). Compared with the reference quartile, the level of 3-PBA in >50th percentile of urine was positively related to the percentage of high DNA fragmentation index. Also, a positive association was observed between the CDCCA 4th percentile and the percentage of medium DNA fragmentation index and the percentage of immature sperms. Only one study particularly focused on sperm aneuploidy. Michał Radwan et al. (83) examined the relationship between exposure to pyrethroids and sperm aneuploidy. With regard to the urinary metabolite of pyrethroids, the levels of TDCCA and CDCCD in urine of the >50th percentile were related to XY disomy and chromosome 18 disomy, respectively. One study focused on the relationship between human sperm sex ratio and environmental endocrine. Joanna Jurewicz et al. (84, 85) found that the level of urinary pyrethroid metabolites was negatively related to Y:X sperm chromosome ratio.

Three studies focused on fecundability after pyrethroid exposure. In the study by Markku Sallmén (86) involving 578 greenhouses workers and their wives, individuals who were exposed to pyrethroids showed decreased fecundability. The exposure to pyrethroids of men working in greenhouses may be associated with reduced fertility. Tan Lifeng et al. (87) conducted a study involving 32 male workers and 22 male administrators. The results showed that high exposure of fenvalerate was associated with the abnormality in sperm quality. Similarity, Hiroki Toshima (88) conducted a study involving 42 Japanese male partners in couples who underwent infertility consultation. A positive association was observed between pyrethroid exposure level and poor semen quality.

## Discussion

Various recent studies assessed the influence of pyrethroid exposure on the male reproductive system. To our knowledge, this is the first systematic review with a meta-analysis that rigorously evaluated the relationship between pyrethroid exposure and male reproductive disorders. Our meta-analysis has an advantage in comparison with narrative reviews. In this study, we conducted rat and mice meta-analyses, including the effects of gestational and lactational exposure to pyrethroids on F1 offspring and the effects of direct exposure on the male reproductive system. Next, we observed a significant decrease in sperm motility, sperm count, serum testosterone, testis weight, and epididymal weight with the exposure of pyrethroid in rats and mice. Interestingly, gestational and lactational exposure of pyrethroids demonstrated similar damage to the male reproductive system.

Over the past decades, male infertility has received an increasing amount of attention worldwide (89). In the past 50 years, evidence of the decreased sperm concentration in European and African males had been explored (90, 91). There were many reasons that accounted for male infertility. For example, alcohol, endocrine disruptors, and environmental pollutants may play an important role in the decline in sperm concentration. The damage caused by the increasing exposure of endocrine disruptors, organic pollutants, heavy metals, and pesticides in daily life on the human body has been increasingly studied. One cohort study (92) showed that exposure to BPA was associated with abnormal sperm tail morphology. Recently, SPs have been widely used all over the world due to their highly toxic effects on insects and are presented in numerous commercial insecticide formulations. As one of the most frequently applied EDCs, pyrethroids primarily entered the body through skin contact, inhalation, or food/water ingestion. And the metabolites of pyrethroids have frequently been detected in urine samples collected from the general population (15).

Through systematic review and meta-analysis of the literature, we have explored the damage to the male reproductive system by seven pyrethroids, including allethrin, bifenthrin, cypermethrin, deltamethrin, fenvalerate, lambda-cyhalothrin, and permethrin. Our results suggested that all seven pyrethroids had an overall negative effect on semen parameters. Each of them demonstrated a negative effect on sperm count, sperm motility, sperm morphology, testis weight, epididymal weight, and serum testosterone. Besides, we also found evidence that gestational and lactational pyrethroids exposure can lead to male reproductive damage in F1 offspring. By conducting a subgroup analysis, we received a more in-depth view. Considering each group individually, different and interesting results can be observed. When comparing the rat and mice groups, we found that pyrethroids had a stronger effect on sperm and serum testosterone in rat groups. When considering the results for different pyrethroids, we found that all pyrethroid types damaged the male reproductive system. Further subgroup analysis demonstrated that sperm count, sperm motility, sperm morphology, testis weight epididymis weight, and serum testosterone showed a consistent trend with SP exposure. A low amount of data was found in some subgroups, and some results were not statistically significant. Next, we also performed a systematic review of human research on the effect of SPs about the male reproductive system. Interestingly, the metabolites of SPs can be detected in the urine of almost everyone. Further studies demonstrated that high-risk groups, such as workers who have been exposed to SPs for a long time, were found to have more SPs metabolites in their urine. Besides, the concentration of SPs metabolites in urine was often negatively correlated with sperm quality.

The mechanisms underlying the male reproductive toxicity of pyrethroids are still being explored. One research demonstrated that bifenthrin reduced sperm motility and kinematic parameters by reducing intracellular ATP level (68). Long term exposure to pyrethroid interferes with the expression of genes that govern spermatogenesis, steroidogenesis, apoptosis, and genetic reprogramming of male gametes (69). To further explore the potential associations between SPs and oxidative stress, we evaluated oxidative stress induction on rats and mice exposed to pyrethroids. The results showed that some pyrethroids, such as lambda-cyhalothrin, deltamethrin, cypermethrin, and bifenthrin can increase the level of MDA oxide in testes and reduce the activities of GSH, SOD, and CAT. And the concentration changes of MDA, GSH, SOD, and CAT were observed in parallel to the reduction in the sperm count. Nano selenium, as a potent antioxidant, had been reported to minimize reproductive toxic effects of SPs by decreasing the concentration of MDA (70). Spirulina, which is a microalga rich in antioxidant compounds, had been found to reverse deleterious effects of the reproductive system caused by bifenthrin. Our assessment provided valuable information for exploring the cause of infertile men. And we hope that our research can provide better understanding for male infertility patients.

## Conclusion

In this study, we provided interesting results from rodent studies regarding the influence of pyrethroids on male reproduction. Based on the aforementioned data, all seven SPs demonstrated toxicity to the male reproductive system. SP exposure during pregnancy and adolescence can cause damage to male F1 offspring or the male reproductive system. Oxidative stress may be essential in the damage of the male reproductive system. Finally, due to the wide use of SPs, humans will inevitably be widely exposed to SPs. And SP exposure was closely associated with human sperm quality.

## Author Contributions

XZ, XC and TZ collected the data and performed the meta-analysis. XR and XZ wrote the manuscript. All authors contributed to the article and approved the submitted version.

## Funding

This work was supported by the National Natural Science Foundation of China (81972386, 81672531 to CQ).

## Conflict of Interest

The authors declare that the research was conducted in the absence of any commercial or financial relationships that could be construed as a potential conflict of interest.

## References

[1] Katsuda Y. Progress and Future of Pyrethroids. Top Curr Chem (2012) 314:1–30. 10.1007/128_2011_252 22048685

[2] Mehrpour O Karrari P Zamani N Tsatsakis AM Abdollahi M. Occupational Exposure to Pesticides and Consequences on Male Semen and Fertility: A Review. Toxicol Lett (2014) 230(2):146–56. 10.1016/j.toxlet.2014.01.029 24487096

[3] Saillenfait AM Ndiaye D Sabate JP. Pyrethroids: Exposure and Health Effects–an Update. Int J Hygiene Environ Health (2015) 218(3):281–92. 10.1016/j.ijheh.2015.01.002 25648288

[4] Freemont JA Littler SW Hutt OE Mauger S Meyer AG Winkler DA. Molecular Markers for Pyrethrin Autoxidation in Stored Pyrethrum Crop: Analysis and Structure Determination. J Agric Food Chem (2016) 64(38):7134–41. 10.1021/acs.jafc.6b02959 27599033

[5] Saillenfait AM Ndiaye D Sabate JP. The Estrogenic and Androgenic Potential of Pyrethroids In Vitro. Review. Toxicol Vitro Int J Published Assoc BIBRA (2016) 34:321–32. 10.1016/j.tiv.2016.02.020 26921664

[6] Wang X Martinez MA Dai M Chen D Ares I Romero A. Permethrin-Induced Oxidative Stress and Toxicity and Metabolism. A Review. Environ Res (2016) 149:86–104. 10.1016/j.envres.2016.05.003 27183507

[7] Marettova E Maretta M Legath J. Effect of Pyrethroids on Female Genital System. Review. Anim Reprod Sci (2017) 184:132–8. 10.1016/j.anireprosci.2017.07.007 28735887

[8] Burns CJ Pastoor TP. Pyrethroid Epidemiology: A Quality-Based Review. Crit Rev Toxicol (2018) 48(4):297–311. 10.1080/10408444.2017.1423463 29389244

[9] Chrustek A Holynska-Iwan I Dziembowska I Bogusiewicz J Wroblewski M Cwynar A. Current Research on the Safety of Pyrethroids Used as Insecticides. Medicina (Kaunas Lithuania) (2018) 54(4). 10.3390/medicina54040061 PMC6174339 30344292

[10] Lu Q Sun Y Ares I Anadon A Martinez M Martinez-Larranaga MR. Deltamethrin Toxicity: A Review of Oxidative Stress and Metabolism. Environ Res (2019) 170:260–81. 10.1016/j.envres.2018.12.045 30599291

[11] Matsuo N. Discovery and Development of Pyrethroid Insecticides. Proc Jpn Acad Ser B Phys Biol Sci (2019) 95(7):378–400. 10.2183/pjab.95.027 PMC6766454 31406060

[12] Navarrete-Meneses MDP Perez-Vera P. Pyrethroid Pesticide Exposure and Hematological Cancer: Epidemiological, Biological and Molecular Evidence. Rev Environ Health (2019) 34(2):197–210. 10.1515/reveh-2018-0070 30903760

[13] Ye X Liu J. Effects of Pyrethroid Insecticides on Hypothalamic-Pituitary-Gonadal Axis: A Reproductive Health Perspective. Environ pollution (Barking Essex 1987) (2019) 245:590–9. 10.1016/j.envpol.2018.11.031 30476888

[14] Lybrand DB Xu H Last RL Pichersky E. How Plants Synthesize Pyrethrins: Safe and Biodegradable Insecticides. Trends Plant Sci (2020) 25(12):1240–51. 10.1016/j.tplants.2020.06.012 PMC7677217 32690362

[15] Shi X-D Bi H-J Fu H-L Li L-Y Liu D-K Li M-J. Effect of Low-Dose Fenvalerate on Semen Quality Capacitation in Adult Mice. Chin Med J (English Edition) (2011) 124(10):1529–33. 10.3760/cma.j.issn.0366-6999.2011.10.017 21740811

[16] Bhunya SP Pati PC. Effect of Deltamethrin, a Synthetic Pyrethroid, on the Induction of Chromosome Aberrations, Micronuclei and Sperm Abnormalities in Mice. Mutagenesis (1990) 5(3):229–32. 10.1093/mutage/5.3.229 2385176

[17] Elbetieha A Da’as SI Khamas W Darmani H. Evaluation of the Toxic Potentials of Cypermethrin Pesticide on Some Reproductive and Fertility Parameters in the Male Rats. Arch Environ Contamination Toxicol (2001) 41(4):522–8. 10.1007/s002440010280 11598791

[18] Hu J-Y Wang S-L Zhao R-C Yang J Chen J-H Song L. [Effects of Fenvalerate on Reproductive and Endocrine Systems of Male Rats]. Zhonghua Nan Ke Xue = Natl J Andrology (2002) 8(1):18–21. 12479040

[19] Mani U Islam F Prasad AK Kumar P Suresh Kumar V Maji BK. Steroidogenic Alterations in Testes and Sera of Rats Exposed to Formulated Fenvalerate by Inhalation. Hum Exp Toxicol (2002) 21(11):593–7. 10.1191/0960327102ht298oa 12507254

[20] Xu LC Zhan NY Liu R Song L Wang XR. Joint Action of Phoxim and Fenvalerate on Reproduction in Male Rats. Asian J Andrology (2004) 6(4):337–41. 15546026

[21] Song L Wang YB Sun H Gu AH Sun Y Wang XR. [Fenvalerate Affects Sperm Motility in SD Rats]. Zhonghua Nan Ke Xue = Natl J Andrology (2007) 13(7):588–91. 17725298

[22] Zhang SY Ito Y Yamanoshita O Yanagiba Y Kobayashi M Taya K. Permethrin may Disrupt Testosterone Biosynthesis Via Mitochondrial Membrane Damage of Leydig Cells in Adult Male Mouse. Endocrinology (2007) 148(8):3941–9. 10.1210/en.2006-1497 17463061

[23] Arena AC Fernandez CD Porto EM Bissacot DZ Pereira OC Kempinas WG. Fenvalerate, a Pyrethroid Insecticide, Adversely Affects Sperm Production and Storage in Male Rats. J Toxicol Environ Health Part A (2008) 71(23):1550–8. 10.1080/15287390802392024 18923997

[24] Hu JY Wang XR. [Joint Action of Phoxim and Fenvalerate on Spermatogenesis of Male Rats]. Zhonghua Nan Ke Xue = Natl J Andrology (2008) 14(11):968–72. 19102494

[25] Song L Wang YB Sun H Yuan C Hong X Qu JH. Effects of Fenvalerate and Cypermethrin on Rat Sperm Motility Patterns In Vitro as Measured by Computer-Assisted Sperm Analysis. J Toxicol Environ Health Part A (2008) 71(5):325–32. 10.1080/15287390701738517 18214806

[26] Zhang SY Ueyama J Ito Y Yanagiba Y Okamura A Kamijima M. Permethrin may Induce Adult Male Mouse Reproductive Toxicity Due to Cis Isomer Not Trans Isomer. Toxicology (2008) 248(2-3):136–41. 10.1016/j.tox.2008.03.018 18455858

[27] Issam C Samir H Zohra H Monia Z Hassen BC. Toxic Responses to Deltamethrin (DM) Low Doses on Gonads, Sex Hormones and Lipoperoxidation in Male Rats Following Subcutaneous Treatments. J Toxicol Sci (2009) 34(6):663–70. 10.2131/jts.34.663 19952501

[28] Wang XZ Liu SS Sun Y Wu JY Zhou YL Zhang JH. Beta-Cypermethrin Impairs Reproductive Function in Male Mice by Inducing Oxidative Stress. Theriogenology (2009) 72(5):599–611. 10.1016/j.theriogenology.2009.04.016 19500828

[29] Abdallah FB Hamden K Galeraud-Denis I El Feki A Keskes-Ammar L. An In Vitro Study on Reproductive Toxicology of Deltamethrin on Rat Spermatozoa. Andrologia (2010) 42(4):254–9. 10.1111/j.1439-0272.2009.00986.x 20629649

[30] Abdallah FB Slima AB Dammak I Keskes-Ammar L Mallek Z. Comparative Effects of Dimethoate and Deltamethrin on Reproductive System in Male Mice. Andrologia (2010) 42(3):182–6. 10.1111/j.1439-0272.2009.00976.x 20500747

[31] Al-Hamdani NM Yajurvedi HN. Cypermethrin Reversibly Alters Sperm Count Without Altering Fertility in Mice. Ecotoxicol Environ Saf (2010) 73(5):1092–7. 10.1016/j.ecoenv.2010.04.009 20435348

[32] Perobelli JE Martinez MF da Silva Franchi CA Fernandez CDB Viana de Camargo JL Kempinas WDG. Decreased Sperm Motility in Rats Orally Exposed to Single or Mixed Pesticides. J Toxicol Environ Health-Part a-Current Issues (2010) 73(13-14):991–1002. 10.1080/15287391003751802 20563933

[33] Prakash N Vijay KM Sunilchandra U Pavithra B Pawar A. Evaluation of Testicular Toxicity Following Short-Term Exposure to Cypermethrin in Albino Mice. Toxicol Int (2010) 17(1):18–21. 10.4103/0971-6580.68344 21042468 PMC2964742

[34] Wang H Wang Q Zhao XF Liu P Meng XH Yu T. Cypermethrin Exposure During Puberty Disrupts Testosterone Synthesis Via Downregulating StAR in Mouse Testes. Arch Toxicol (2010) 84(1):53–61. 10.1007/s00204-009-0479-y 19862501

[35] Yuan C Wang C Gao SQ Kong TT Chen L Li XF. Effects of Permethrin, Cypermethrin and 3-Phenoxybenzoic Acid on Rat Sperm Motility In Vitro Evaluated With Computer-Assisted Sperm Analysis. Toxicol Vitro Int J Published Assoc BIBRA (2010) 24(2):382–6. 10.1016/j.tiv.2009.11.001 19896529

[36] Zhang H Wang H Wang Q Zhao XF Liu P Ji YL. Pubertal and Early Adult Exposure to Fenvalerate Disrupts Steroidogenesis and Spermatogenesis in Mice at Adulthood. J Appl Toxicol JAT (2010) 30(4):369–77. 10.1002/jat.1507 20063364

[37] Issam C Zohra H Monia Z Hassen BC. Effects of Dermal Sub-Chronic Exposure of Pubescent Male Rats to Permethrin (PRMT) on the Histological Structures of Genital Tract, Testosterone and Lipoperoxidation. Exp Toxicologic Pathol (2011) 63(4):393–400. 10.1016/j.etp.2010.02.016 20381324

[38] Joshi SC Bansal B Jasuja ND. Evaluation of Reproductive and Developmental Toxicity of Cypermethrin in Male Albino Rats. Toxicol Environ Chem (2011) 93(3):593–602. 10.1080/02772248.2010.537441

[39] Ben Abdallah F Fetoui H Zribi N Fakhfakh F Keskes L. Protective Role of Caffeic Acid on Lambda Cyhalothrin-Induced Changes in Sperm Characteristics and Testicular Oxidative Damage in Rats. Toxicol Ind Health (2012) 28(7):639–47. 10.1177/0748233711420470 22025501

[40] Gao X Wang Q Wang J Wang C Lu L Gao R. Expression of Calmodulin in Germ Cells is Associated With Fenvalerate-Induced Male Reproductive Toxicity. Arch Toxicol (2012) 86(9):1443–51. 10.1007/s00204-012-0825-3 PMC3529155 22437841

[41] Li X Cai D. [Single and Combined Toxic Effects of di-2-ethylhexyl Phthalate and Cypermethrin on Fertility and Development in the the Prepubertal Male Rats]. Wei Sheng Yan Jiu = J Hygiene Res (2012) 41(5):710–6. 23213681

[42] Oda SS El-Maddawy Z. Protective Effect of Vitamin E and Selenium Combination on Deltamethrin-Induced Reproductive Toxicity in Male Rats. Exp Toxicologic Pathol Off J Gesellschaft fur Toxikologische Pathologie (2012) 64(7-8):813–9. 10.1016/j.etp.2011.03.001 21478004

[43] Sakr SA Al-Amoudi WM. Effect of Ginger Extract on Deltamethrin Induced Histomorphological and Immunohistochemical Changes in Testes of Albino Rats. Life Sci Journal-Acta Zhengzhou Univ Overseas Edition (2012) 9(1):771–8.

[44] Wang D Kamijima M Okamura A Ito Y Yanagiba Y X-f J. Evidence for Diazinon-Mediated Inhibition of Cis-Permethrin Metabolism and its Effects on Reproductive Toxicity in Adult Male Mice. Reprod Toxicol (2012) 34(4):489–97. 10.1016/j.reprotox.2012.07.007 22944209

[45] Ben Abdallah F Fetoui H Zribi N Fakhfakh F Keskes L. Quercetin Attenuates Lambda Cyhalothrin-Induced Reproductive Toxicity in Male Rats. Environ Toxicol (2013) 28(12):673–80. 10.1002/tox.20762 21887817

[46] Ben Slima A Ali MB Barkallah M Traore AI Boudawara T Allouche N. Antioxidant Properties of Pelargonium Graveolens L’Her Essential Oil on the Reproductive Damage Induced by Deltamethrin in Mice as Compared to Alpha-Tocopherol. Lipids Health Dis (2013) 12:30. 10.1186/1476-511X-12-30 23496944 PMC3641007

[47] Hu JX Li YF Li J Pan C He Z Dong HY. Toxic Effects of Cypermethrin on the Male Reproductive System: With Emphasis on the Androgen Receptor. J Appl Toxicol JAT (2013) 33(7):576–85. 10.1002/jat.1769 22147539

[48] Li Yan F Pan C Hu Jin X Li J Xu Li C. Effects of Cypermethrin on Male Reproductive System in Adult Rats. Biomed Environ Sci (2013) 26(3):201–8. 10.3967/0895-3988.2013.03.007 23425803

[49] Sharma P Huq AU Singh R. Cypermethrin Induced Reproductive Toxicity in Male Wistar Rats: Protective Role of Tribulus Terrestris. J Environ Biol (2013) 34(5):857–62. 24558798

[50] Al-Sarar AS Abobakr Y Bayoumi AE Hussein HI Al-Ghothemi M. Reproductive Toxicity and Histopathological Changes Induced by Lambda-Cyhalothrin in Male Mice. Environ Toxicol (2014) 29(7):750–62. 10.1002/tox.21802 22865375

[51] Ben Halima N Ben Slima A Moalla I Fetoui H Pichon C Gdoura R. Protective Effects of Oat Oil on Deltamethrin-Induced Reprotoxicity in Male Mice. Food Funct (2014) 5(9):2070–7. 10.1039/C4FO00190G 24992227

[52] Madhubabu G Yenugu S. Allethrin Induced Toxicity in the Male Reproductive Tract of Rats Contributes to Disruption in the Transcription of Genes Involved in Germ Cell Production. Environ Toxicol (2014) 29(11):1330–45. 10.1002/tox.21864 23595975

[53] Sharma P Huq AU Singh R. Cypermethrin-Induced Reproductive Toxicity in the Rat is Prevented by Resveratrol. J Hum Reprod Sci (2014) 7(2):99–106. 10.4103/0974-1208.138867 25191022 PMC4150150

[54] Sharma P Singh R Jan M. Dose-Dependent Effect of Deltamethrin in Testis, Liver, and Kidney of Wistar Rats. Toxicol Int (2014) 21(2):131–9. 10.4103/0971-6580.139789 PMC4170553 25253921

[55] Mostafa Hel S Abd El-Baset SA Kattaia AA Zidan RA Al Sadek MM. Efficacy of Naringenin Against Permethrin-Induced Testicular Toxicity in Rats. Int J Exp Pathol (2016) 97(1):37–49. 10.1111/iep.12168 26867500 PMC4840246

[56] Patrick SM Bornman MS Joubert AM Pitts N Naidoo V de Jager C. Effects of Environmental Endocrine Disruptors, Including Insecticides Used for Malaria Vector Control on Reproductive Parameters of Male Rats. Reprod Toxicol (Elmsford NY) (2016) 61:19–27. 10.1016/j.reprotox.2016.02.015 26928317

[57] El Sayed Mostafa H El-Baset SAA Kattaia AAA Zidan RA Al Sadek MMA. Efficacy of Naringenin Against Permethrin-Induced Testicular Toxicity in Rats. Int J Exp Pathol (2016) 97(1):37–49. 10.1111/iep.12168 26867500 PMC4840246

[58] Alaa-Eldin EA El-Shafei DA Abouhashem NS. Individual and Combined Effect of Chlorpyrifos and Cypermethrin on Reproductive System of Adult Male Albino Rats. Environ Sci Pollution Res Int (2017) 24(2):1532–43. 10.1007/s11356-016-7912-6 27785720

[59] Khaki A Khaki AA Rajabzadeh A. The Effects of Permethrin and Antioxidant Properties of Allium Cepa (Onion) on Testicles Parameters of Male Rats. Toxin Rev (2017) 36(1):1–6. 10.1080/15569543.2016.1235582

[60] Madhubabu G Yenugu S. Allethrin Toxicity Causes Reproductive Dysfunction in Male Rats. Environ Toxicol (2017) 32(6):1701–10. 10.1002/tox.22394 28181402

[61] Sharma P Aslam Khan I Singh R. Curcumin and Quercetin Ameliorated Cypermethrin and Deltamethrin-Induced Reproductive System Impairment in Male Wistar Rats by Upregulating the Activity of Pituitary-Gonadal Hormones and Steroidogenic Enzymes. Int J Fertility Sterility (2018) 12(1):72–80. 10.1055/s-0034-1382471 PMC5767937 29334211

[62] Zhang J Hu Y Guo J Pan R Shi R Tian Y. Fenvalerate Decreases Semen Quality in Puberty Rat Through Germ Cell Apoptosis. Andrologia (2018) 50(9):e13079. 10.1111/and.13079 29968265

[63] Bagherpour H Karimpour Malekshah A Talebpour Amiri F Azadbakht M. Protective Effect of Green Tea Extract on the Deltamethrin-Induced Toxicity in Mice Testis: An Experimental Study. Int J Reprod Biomed (Yazd Iran) (2019) 17(5):337–48. 10.18502/ijrm.v17i5.4601 PMC6653493 31435613

[64] Hong T Li R Sun L-L Xu J He M-T Wang W. Role of the Gene Phlda1 in Fenvalerate-Induced Apoptosis and Testicular Damage in Sprague-Dawley Rats. J Toxicol Environ Health-Part a-Current Issues (2019) 82(15):870–8. 10.1080/15287394.2019.1664584 31524104

[65] Osama E Galal AAA Abdalla H El-Sheikh SMA. Chlorella Vulgaris Ameliorates Testicular Toxicity Induced by Deltamethrin in Male Rats Via Modulating Oxidative Stress. Andrologia (2019) 51(3):e13214. 10.1111/and.13214 30488469

[66] Barkallah M Slima AB Elleuch F Fendri I Pichon C Abdelkafi S. Protective Role of Spirulina Platensis Against Bifenthrin-Induced Reprotoxicity in Adult Male Mice by Reversing Expression of Altered Histological, Biochemical, and Molecular Markers Including Micrornas. Biomolecules (2020) 10(5). 10.3390/biom10050753 PMC7277961 32408700

[67] Maksoud HA Mahfouz M Soliman M Elharrif MG Abbass M El-Badry M. Harmful Effects of Pyrethroid Ester Insecticide on the Male Reproductive System Mainly Through Affecting Testicular Function and Inflammatory Markers. Biocell (2020) 44(1):111–5. 10.32604/biocell.2020.08399

[68] Bae JW Kwon WS. The Deleterious Toxic Effects of Bifenthrin on Male Fertility. Reprod Toxicol (Elmsford NY) (2021) 101:74–80. 10.1016/j.reprotox.2021.03.002 33713777

[69] Ravula AR Yenugu S. Effect of Oral Administration of a Mixture of Pyrethroids at Doses Relevant to Human Exposure on the General and Male Reproductive Physiology in the Rat. Ecotoxicol Environ Saf (2021) 208:111714. 10.1016/j.ecoenv.2020.111714 33396045

[70] Hozyen HF Khalil HMA Ghandour RA Al-Mokaddem AK Amer MS Azouz RA. Nano Selenium Protects Against Deltamethrin-Induced Reproductive Toxicity in Male Rats. Toxicol Appl Pharmacol (2020) 408:115274. 10.1016/j.taap.2020.115274 33038357

[71] Katragadda V Adem M Mohammad RA Sri Bhasyam S Battini K. Testosterone Recuperates Deteriorated Male Fertility in Cypermethrin Intoxicated Rats. Toxicol Res (2021) 37(1):125–34. 10.1007/s43188-020-00046-1 PMC7806673 33489863

[72] Meeker JD Barr DB Hauser R. Human Semen Quality and Sperm DNA Damage in Relation to Urinary Metabolites of Pyrethroid Insecticides. Hum Reprod (2008) 23(8):1932–40. 10.1093/humrep/den242 18579513 PMC2733825

[73] Radwan M Jurewicz J Wielgomas B Sobala W Piskunowicz M Radwan P. Semen Quality and the Level of Reproductive Hormones After Environmental Exposure to Pyrethroids. J Occup Environ Med (2014) 56(11):1113–9. 10.1097/JOM.0000000000000297 25376404

[74] Yoshinaga J Imai K Shiraishi H Nozawa S Yoshiike M Mieno MN. Pyrethroid Insecticide Exposure and Reproductive Hormone Levels in Healthy Japanese Male Subjects. Andrology (2014) 2(3):416–20. 10.1111/j.2047-2927.2014.00202.x 24634311

[75] Xia Y Han Y Wu B Wang S Gu A Lu N. The Relation Between Urinary Metabolite of Pyrethroid Insecticides and Semen Quality in Humans. Fertility Sterility (2008) 89(6):1743–50. 10.1016/j.fertnstert.2007.05.049 17765231

[76] Meeker JD Barr DB Hauser R. Pyrethroid Insecticide Metabolites are Associated With Serum Hormone Levels in Adult Men. Reprod Toxicol (Elmsford NY) (2009) 27(2):155–60. 10.1016/j.reprotox.2008.12.012 PMC2692246 19429394

[77] Hu Y Zhang Y Vinturache A Wang Y Shi R Chen L. Effects of Environmental Pyrethroids Exposure on Semen Quality in Reproductive-Age Men in Shanghai, China. Chemosphere (2020) 245:125580. 10.1016/j.chemosphere.2019.125580 31855762

[78] Santos R Piccoli C Cremonese C Freire C. Thyroid and Reproductive Hormones in Relation to Pesticide Use in an Agricultural Population in Southern Brazil. Environ Res (2019) 173:221–31. 10.1016/j.envres.2019.03.050 30928852

[79] Zalata A Elhanbly S Abdalla H Serria MS Aziz A El-Dakrooy SA. In Vitro Study of Cypermethrin on Human Spermatozoa and the Possible Protective Role of Vitamins C, and XXXE. Andrologia (2014) 46(10):1141–7. 10.1111/and.12206 24329529

[80] Ji G Xia Y Gu A Shi X Long Y Song L. Effects of non-Occupational Environmental Exposure to Pyrethroids on Semen Quality and Sperm DNA Integrity in Chinese Men. Reprod Toxicol (Elmsford NY) (2011) 31(2):171–6. 10.1016/j.reprotox.2010.10.005 20955780

[81] Xia Y Bian Q Xu L Cheng S Song L Liu J. Genotoxic Effects on Human Spermatozoa Among Pesticide Factory Workers Exposed to Fenvalerate. Toxicology (2004) 203(1-3):49–60. 10.1016/j.tox.2004.05.018 15363581

[82] Bian Q Xu LC Wang SL Xia YK Tan LF Chen JF. Study on the Relation Between Occupational Fenvalerate Exposure and Spermatozoa DNA Damage of Pesticide Factory Workers. Occup Environ Med (2004) 61(12):999–1005. 10.1136/oem.2004.014597 15550606 PMC1740696

[83] Radwan M Jurewicz J Wielgomas B Piskunowicz M Sobala W Radwan P. The Association Between Environmental Exposure to Pyrethroids and Sperm Aneuploidy. Chemosphere (2015) 128:42–8. 10.1016/j.chemosphere.2014.12.077 25655817

[84] Jurewicz J Radwan M Sobala W Radwan P Jakubowski L Wielgomas B. Exposure, to Widespread Environmental Endocrine Disrupting Chemicals and Human Sperm Sex Ratio. Environ Pollution (2016) 213:732–40. 10.1016/j.envpol.2016.02.008 27031570

[85] Jurewicz J Radwan M Wielgomas B Sobala W Piskunowicz M Radwan P. The Effect of Environmental Exposure to Pyrethroids and DNA Damage in Human Sperm. Syst Biol Reprod Med (2015) 61(1):37–43. 10.3109/19396368.2014.981886 25376306

[86] Sallmén M Liesivuori J Taskinen H Lindbohm ML Anttila A Aalto L. Time to Pregnancy Among the Wives of Finnish Greenhouse Workers. Scandinavian J Work Environ Health (2003) 29(2):85–93. 10.5271/sjweh.709 12718493

[87] Lifeng T Shoulin W Junmin J Xuezhao S Yannan L Qianli W. Effects of Fenvalerate Exposure on Semen Quality Among Occupational Workers. Contraception (2006) 73(1):92–6. 10.1016/j.contraception.2005.06.067 16371303

[88] Toshima H Suzuki Y Imai K Yoshinaga J Shiraishi H Mizumoto Y. Endocrine Disrupting Chemicals in Urine of Japanese Male Partners of Subfertile Couples: A Pilot Study on Exposure and Semen Quality. Int J Hygiene Environ Health (2012) 215(5):502–6. 10.1016/j.ijheh.2011.09.005 21958682

[89] Johnson SL Dunleavy J Gemmell NJ Nakagawa S. Consistent Age-Dependent Declines in Human Semen Quality: A Systematic Review and Meta-Analysis. Ageing Res Rev (2015) 19:22–33. 10.1016/j.arr.2014.10.007 25462195

[90] Sengupta P Borges E Dutta S Krajewska-Kulak E. Decline in Sperm Count in European Men During the Past 50 Years. Hum Exp Toxicol (2017) 37(3):247–55. 10.1177/0960327117703690 28413887

[91] Sengupta P Nwagha U Dutta S Krajewska-Kulak E Izuka E. Evidence for Decreasing Sperm Count in African Population From 1965 to 2015. Afr Health Sci (2017) 17(2):418–27. 10.4314/ahs.v17i2.16 PMC5637027 29062337

[92] Pollard SH Cox KJ Blackburn BE Wilkins DG Carrell DT Stanford JB. Male Exposure to Bisphenol A (BPA) and Semen Quality in the Home Observation of Periconceptional Exposures (HOPE) Cohort. Reprod Toxicol (Elmsford NY) (2019) 90:82–7. 10.1016/j.reprotox.2019.08.014 PMC6885548 31445078

