# Supplementary figures and images for: Pyrethroids Toxicity to Male Reproductive System and Offspring as a Function of Oxidative Stress Induction: Rodent Studies
Source: Front Endocrinol (Lausanne). 2021 May 27;12:656106. doi: 10.3389/fendo.2021.656106 (PMC8190395; doi:10.3389/fendo.2021.656106)

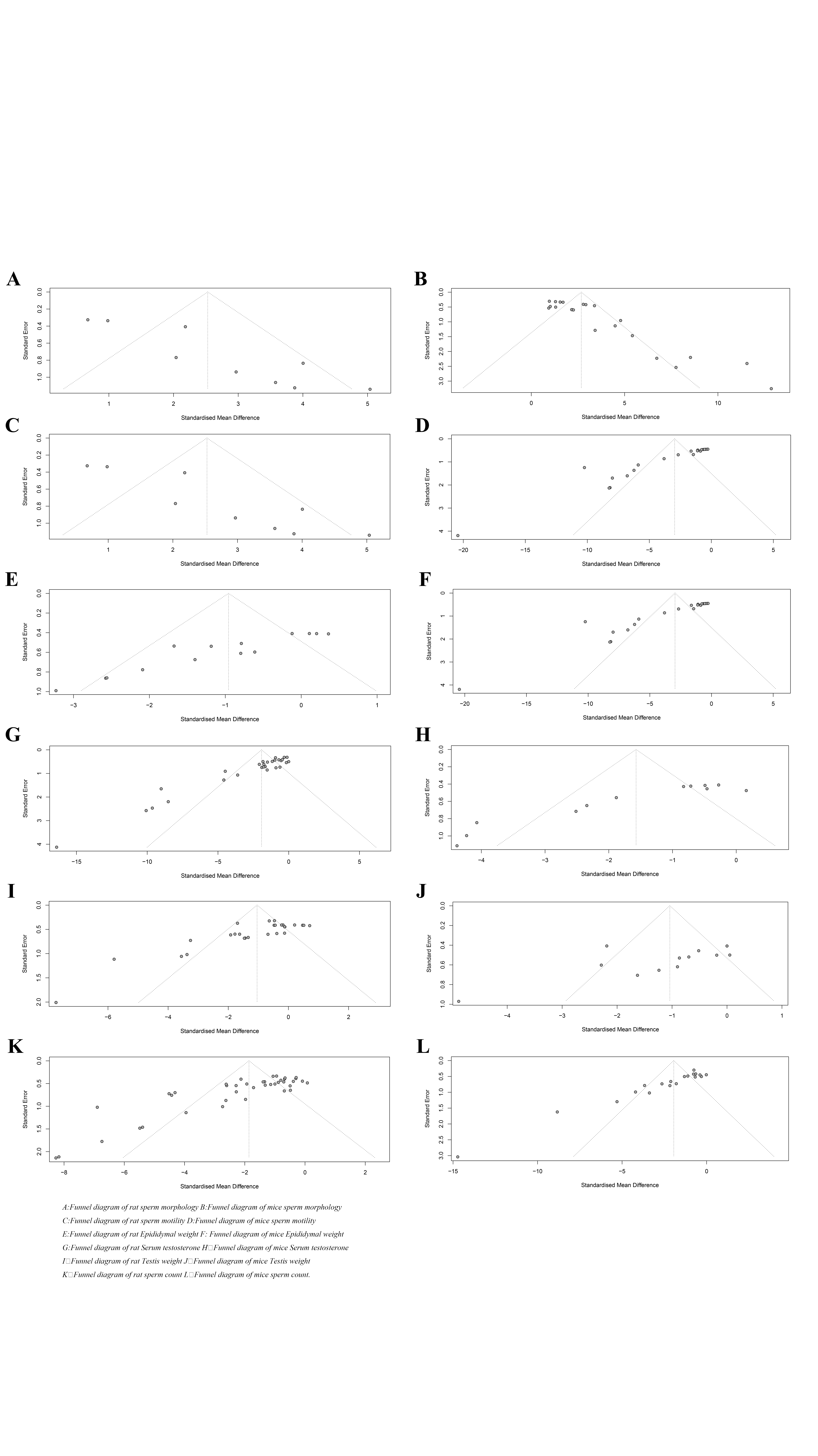

Supplement: Supplementary file 1 [file Image_1.png]
